# Inefficient induction of circulating TAA-specific CD8+ T-cell responses in hepatocellular carcinoma

**DOI:** 10.18632/oncotarget.27146

**Published:** 2019-08-27

**Authors:** Catrin Tauber, Michael Schultheiss, Raffaele De Luca, Nico Buettner, Sian Llewellyn-Lacey, Florian Emmerich, Sebastian Zehe, David A. Price, Christoph Neumann-Haefelin, Annette Schmitt-Graeff, Maike Hofmann, Robert Thimme

**Affiliations:** ^1^ Department of Medicine II, University Hospital Freiburg - Faculty of Medicine, University of Freiburg, Freiburg, Germany; ^2^ Faculty of Biology, University of Freiburg, Freiburg, Germany; ^3^ Division of Infection and Immunity, Cardiff University School of Medicine, Cardiff, United Kingdom; ^4^ Institute for Cell and Gene Therapy, University Hospital Freiburg - Faculty of Medicine, University of Freiburg, Freiburg, Germany; ^5^ Institute of Pathology, University Hospital Freiburg - Faculty of Medicine, University of Freiburg, Freiburg, Germany

**Keywords:** TAA, MAGE-A, HCC, T-cell exhaustion, liver cirrhosis

## Abstract

**Background & Aims:** In hepatocellular carcinoma (HCC), CD8+ T-cell responses targeting tumor-associated antigens (TAA) are considered to be beneficial. However, the molecular profile of TAA-specific CD8+ T cells in HCC is not well defined due to their low frequency.

**Results:** In this study, we demonstrate that TAA-specific CD8+ T-cell responses are not efficiently induced in the peripheral blood of HCC patients as supported by the following observations: First, in HCC patients, frequencies of TAA-specific CD8+ T cells were not increased compared to healthy donors (HD) or patients with liver cirrhosis. Second, a remarkable proportion of TAA-specific CD8+ T cells were naïve despite the presence of antigen within the tumor tissue. Third, antigen-experienced TAA-specific CD8+ T cells lack the characteristic transcriptional regulation of exhausted CD8+ T cells, namely Eomes^hi^Tbet^dim^, and express inhibitory receptors only on a minor proportion of cells. This suggests restricted antigen recognition and further supports the hypothesis of inefficient induction and activation.

**Methods:** By applying peptide/MHCI tetramer-based enrichment, a method of high sensitivity, we now could define the heterogeneity of circulating TAA-specific CD8+ T cells targeting glypican-3, NY-ESO-1, MAGE-A1 and MAGE-A3. We focused on therapy-naïve HCC patients of which the majority underwent transarterial chemoembolization (TACE).

**Conclusion:** Our analysis reveals that circulating TAA-specific CD8+ T cells targeting 4 different immunodominant epitopes are not properly induced in therapy-naïve HCC patients thereby unravelling new and unexpected insights into TAA-specific CD8+ T-cell biology in HCC. This clearly highlights severe limitations of these potentially anti-tumoral T cells that may hamper their biological and clinical relevance in HCC.

## INTRODUCTION

Hepatocellular carcinoma (HCC) is the most common type of primary liver cancer in adults and occurs predominantly in patients with underlying chronic liver disease and cirrhosis. It has a poor prognosis and therapeutic options are limited [1]. There is growing interest to treat HCC with immunotherapeutic approaches as several studies have indicated a role of innate and adaptive immunity in HCC progression and control. For example, lymphocytic infiltrates, in particular tumor-infiltrating T cells that occur during natural disease progression or that are induced by different therapies, have been shown to be associated with improved relapse-free survival [2–4]. In addition, the PD1 inhibitor nivolumab has recently shown durable objective responses in patients with advanced HCC further indicating a role of adaptive immunity, especially of T cells in HCC progression versus control [5].

The tumor antigens recognized by T cells have not been well characterized and may consist of immunogenic neoantigens that have not been identified yet in HCC. However, several TAAs have been shown to be expressed in human HCC [6–13]. TAAs comprise a range of self-derived proteins, such as a-fetoprotein (AFP), glypican-3 (GPC-3), New York esophageal squamous cell carcinoma (NY-ESO-1) or the melanoma-associated gene-A (MAGE-A) that can become immunogenic in HCC either by mutation or aberrant expression. TAAs show different expression rates with e. g. MAGE-A being expressed in up to 80% and NY-ESO-1 being expressed in less than 50% of HCC patients [14–16]. Importantly, there is growing evidence that these TAAs can be recognized by specific CD8+ T-cell responses. Indeed, CD8+ T-cell responses targeting different TAAs have been reported in several studies. Collectively, the results of these studies have given important insights into HCC immunobiology that can be summarized as follows: First, the overall frequency of TAA-specific CD8+ T-cell responses is very low and most T-cell responses were only detectable after antigen-specific [6, 17] or -unspecific [7, 14] expansion. Thus, it has been postulated that the overall amount of TAA-specific CD8+ T cells might be too low to eliminate HCC. Second, however TAA-specific CD8+ T-cell responses have been shown to be associated with improved survival indicating at least a partial biological relevant activity despite their overall low frequency. Third, TAA-specific CD8+ T cells are impaired in their effector functions [18], e. g. showing a reduced TAA-specific cytokine production or killing capacity. The mechanisms responsible for this TAA-specific CD8+ T-cell dysfunction are not well understood but several factors e. g. the expression of inhibitory receptors such as PD-1 [18–20], the immunosuppressive tumor microenvironment [21, 22], the lack of sufficient CD4+ T-cell help [18, 19] or the action of suppressive cytokines [21], regulatory T cells or myeloid derived suppressor cells [17, 19, 21, 22] have been suggested to contribute.

Despite all these important insights into TAA-specific CD8+ T-cell immunity, their low frequency and thus the requirement for *in vitro* expansion for proper T-cell analysis has hampered the analysis of the *ex vivo* molecular properties of TAA-specific CD8+ T cells in HCC. Indeed, only a few studies have analyzed the TAA-specific CD8+ T-cell responses *ex vivo* by pMHCI-tetramers and were also limited by the small amount of detectable cells [20, 23]. Thus, little is known about the *ex vivo* frequency of TAA-specific CD8+ T cells, their differentiation status, e. g. expression of exhaustion markers, their association with antigen expression and response to conventional HCC therapy. Here, by performing pMHCI-tetramer-based enrichment that allows the detection and characterization of rare antigen-specific CD8+ T-cell populations as well as an estimation of their frequency, we set out to address these important questions. Noteworthy, by using this sensitive approach, we were previously able to define key characteristics of HCV-specific CD8+ T cells [24, 25]. In this study, we show that circulating TAA-specific CD8+ T cells are indeed present at very low frequencies even after applying high-sensitivity pMHCI-tetramer-based enrichment probably due to inefficient TAA-specific CD8+ T-cell induction in HCC patients. In line with this, we observed circulating TAA-specific CD8+ T cells with a naïve phenotype and the absence of exhausted TAA-specific CD8+ T cells, both indicative of inefficient activation and restricted antigen recognition. Thus, this comprehensive analysis gives important novel insights into circulating TAA-specific CD8+ T-cell responses in HCC and clearly highlights severe limitations of these potentially anti-tumoral T cells that may hamper their biological and clinical relevance.

## RESULTS

### pMHCI-tetramer enrichment reveals comparable detection rate and frequency of circulating TAA-specific CD8+ T cells in healthy donors, patients with liver cirrhosis and HCC patients

**Figure 1:**
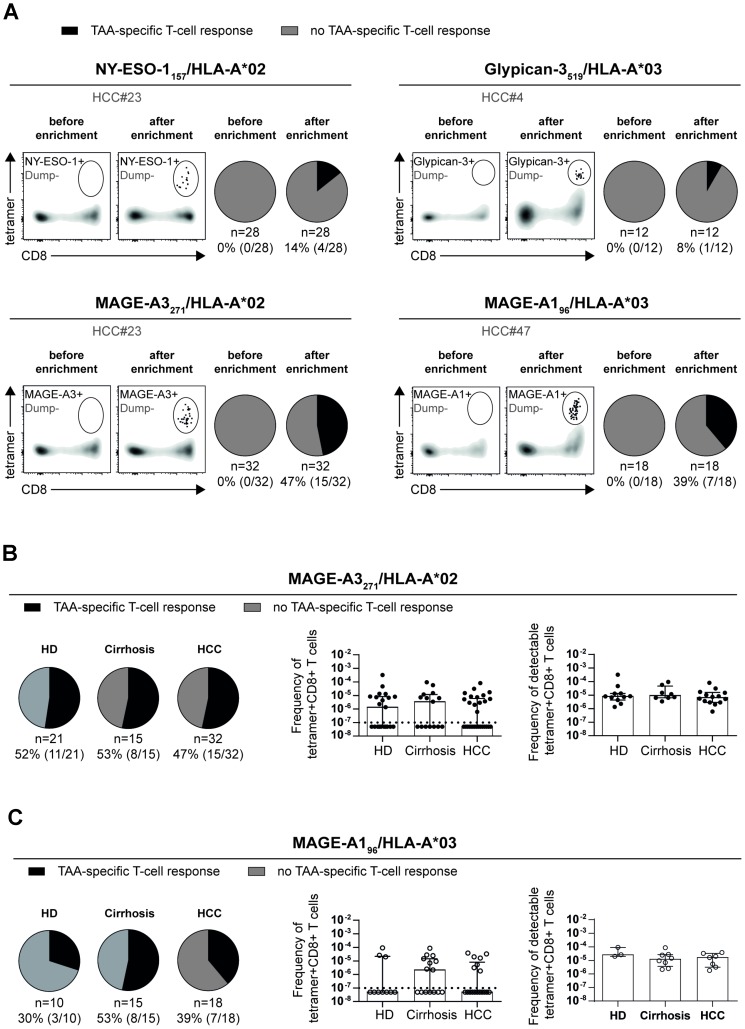
Different detection rates and frequencies of circulating TAA-specific CD8+ T cells. Detection rates of circulating TAA-specific CD8+ T-cell responses targeting NY-ESO-1_157_/HLA-A*02, Glypican-3_519_/HLA-A*03, MAGE-A3_271_/HLA-A*02 and MAGE-A1_96_/HLA-A*03 differ in HCC patients. Representative flow cytometry plots are displayed and pie charts depicting absence (grey) and presence (black) of detectable TAA-specific T-cell responses (**A**). Detection rates, frequencies of all enriched and of detectable MAGE-A3_271_- and MAGE-A1_96_-specific CD8+ T cells in healthy donors, patients with liver cirrhosis or HCC are depicted (**B**, **C**). Dotted line indicates limit of detection (10^−7^ [37];). Statistical analysis was performed using binomial (A–C) test and non-parametric Kruskal-Wallis test (B, C).

In a first set of experiments, we performed pMHCI-tetramer-based enrichment to screen a cohort of 47 therapy-naïve HCC patients (Supplementary Table 1) for the presence of circulating TAA-specific CD8+ T cells targeting the HLA-A*02-restricted epitopes NY-ESO-1_157_, MAGE-A3_271_, Glypican-3_521_ and AFP_47_, and the HLA-A*03-restricted epitopes MAGE-A1_96_, and Glypican-3_519_. This approach was used to increase the detection rate of circulating TAA-specific CD8+ T-cell responses that have been previously reported to be very low [6, 7, 14]. Indeed, by conventional *ex vivo* pMHCI-tetramer staining, we failed to detect any TAA-specific CD8+ T cells. By using the pMHCI-tetramer-based enrichment strategy, it turned out that Glypican-3- and AFP-specific CD8+ T cells could not be reliably enriched using Glypican-3_521_/HLA-A*02 and AFP_47_/HLA-A*02 tetramers (data not shown). Furthermore, only a minority of HCC patients displayed detectable CD8+ T-cell responses against the HLA-A*02-restricted NY-ESO-1_157_ (14%) and HLA-A*03-restricted Glypican-3_519_ (8%) epitopes. However, 15 out of 32 HCC patients (47%) showed a CD8+ T-cell response against the HLA-A*02-restricted MAGE-A3_271_ and 7 out of 18 HCC patients (39%) a response against the HLA-A*03-restricted MAGE-A1_96_ epitope (Figure 1A). Overall, this is a rather low detection rate since by using the same approach we were previously able to detect HCV-specific CD8+ T-cell responses in the majority of chronically infected patients [24]. Thus, these results show that circulating TAA-specific CD8+ T-cell responses are rarely detectable despite applying high-sensitivity techniques like pMHCI-tetramer enrichment.

To determine whether circulating TAA-specific CD8+ T-cell responses are specific for cancer patients, in this case for HCC, we next compared their detection rates and frequencies in HCC patients with the detectability in healthy donors (HD; *n* = 28; Supplementary Table 2) and in patients with liver cirrhosis (*n* = 29; Supplementary Table 3). For this analysis, based on the very low detection rates of NY-ESO_157_- and Glypican-3_519_-specific CD8+ T-cell responses, we only focused on MAGE-A3_271_- and MAGE-A1_96_-specific T cells. Importantly, as shown in Figure 1B, MAGE-A3_271_-specific CD8+ T cells showed a similar detection rate and frequency in all three tested cohorts. In the case of MAGE-A1_96_-specific CD8+ T-cell responses, the detection rate was somewhat, but not significantly, lower in HD compared to the HCC and liver cirrhosis cohorts (30% versus 53% (cirrhosis), and 39% (HCC)), however, the frequencies of detectable CD8+ T-cell responses were in the same range (Figure 1C). Taken together, these results indicate a comparable presence of circulating TAA-specific CD8+ T cells in the different cohorts and surprisingly the absence of a specific expansion of TAA-specific CD8+ T cells in HCC patients.

### Antigen-experienced TAA-specific CD8+ T cells are present in HCC with a high inter-individual variability

**Figure 2:**
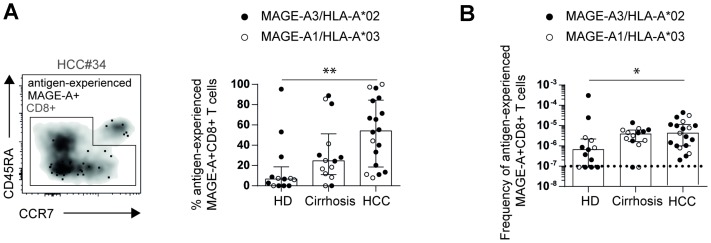
Increased frequencies of circulating antigen-experienced MAGE-A-specific CD8+ T cells in HCC patients. Representative flow cytometry plot, percentages (**A**) and frequencies (**B**) of circulating antigen-experienced MAGE-A-specific CD8+ T cells in HCC patients compared to healthy donors and patients with liver cirrhosis are depicted. Statistical analysis was performed using non-parametric Kruskal-Wallis test (^*^ *p* < 0.005, ^**^ *p* < 0.001).

The low frequency and lack of expansion of circulating TAA-specific CD8+ T cells prompted us to address the question whether these cells were indeed antigen-experienced and had thus been primed and activated *in vivo*. For this analysis, pMHCI-tetramer-enriched MAGE-A1_96_- and MAGE-A3_271_-specific CD8+ T cells were analyzed for the expression of CCR7 and CD45RA. Antigen-experienced cells are defined by a phenotype being either CD45RA+CCR7-, CD45RA-CCR7- or CD45RA-CCR7+ whereas naïve cells are defined by a CD45RA+CCR7+ phenotype. Of note, as shown in Figure 2A and 2B, we found substantial differences in the proportion and frequency of antigen-experienced MAGE-A-specific CD8+ T cells between the different cohorts. As expected, in HD, circulating MAGE-A-specific CD8+ T cells largely displayed a naïve CD45RA+CCR7+ phenotype suggesting that these cells have not been primed *in vivo* (% antigen-experienced MAGE-A-specific CD8+ T cells in HD: Median: 7.0%; IQR: 23.6%). In contrast, circulating MAGE-A-specific CD8+ T cells obtained from HCC patients largely consisted of antigen-experienced cells, however, with a high inter-individual variability (HCC: Median: 52.9%; IQR: 60.8%). Indeed, some HCC patients displayed MAGE-A-specific CD8+ T-cell responses with an almost exclusive naïve phenotype, indicating that these circulating cells may not have been primed *in vivo* or may exhibit a naïve-like phenotype due to abortive activation.

### Lower frequency of antigen-experienced TAA- versus virus-specific CD8+ T cells in HCC patients

**Figure 3:**
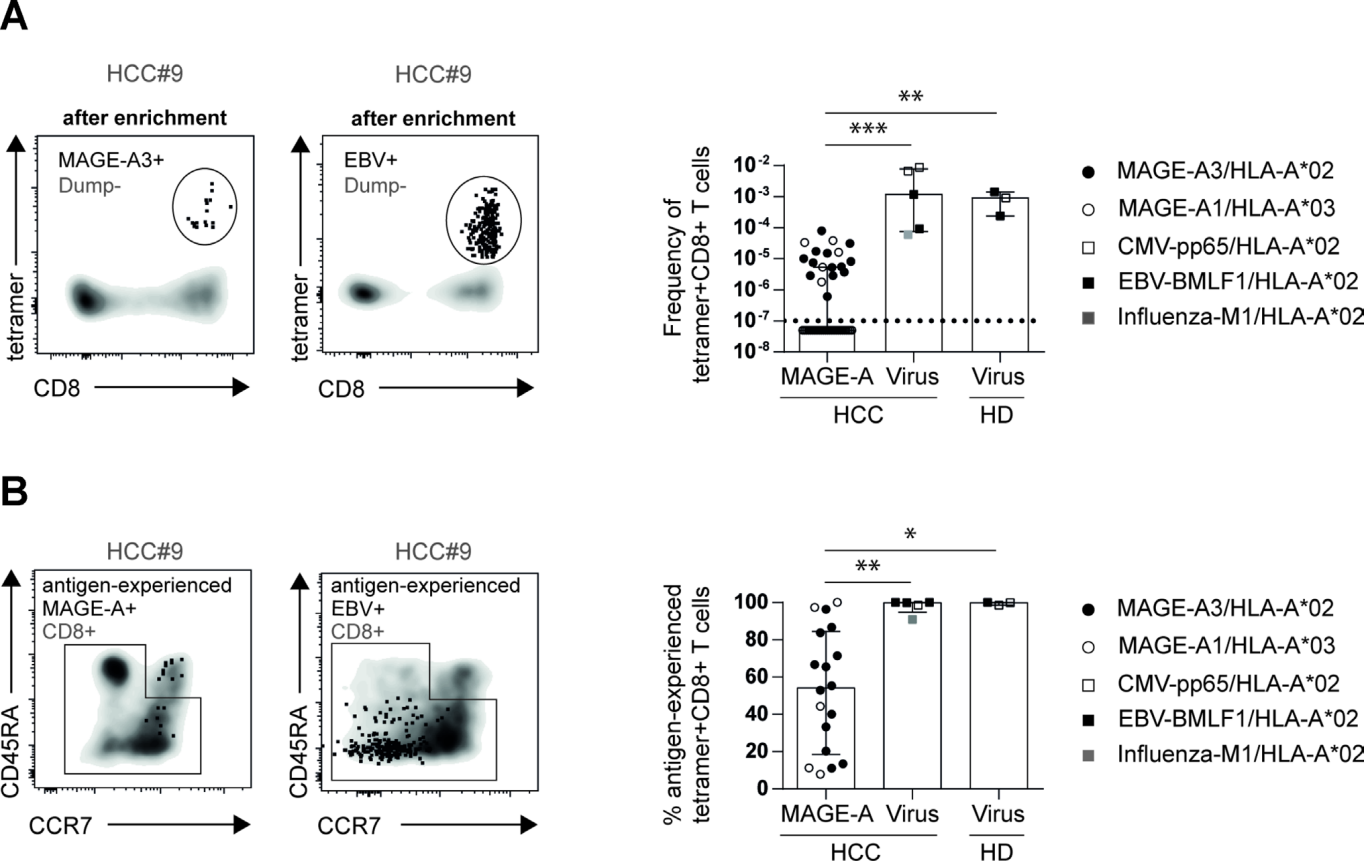
Frequencies of circulating antigen-experienced virus-specific CD8+ T cells are comparable in healthy donors and HCC patients. Representative flow cytometry plots, frequencies (**A**) and percentages of circulating antigen-experienced (**B**) MAGE-A-specific and virus-specific CD8+ T cells are depicted. Statistical analysis was performed using non-parametric Kruskal-Wallis test (^*^ *p* < 0.005, ^**^ *p* < 0.001).

Next, we asked whether the low frequency of circulating TAA-specific CD8+ T cells and their highly variable antigen-experienced phenotype may constitute a general feature of antigen-specific CD8+ T-cell responses present in HCC patients. To address this question, we analyzed MAGE-A-specific and virus-specific CD8+ T cells targeting Influenza, CMV and EBV in the blood of HCC patients. As shown in Figure 3A, virus-specific CD8+ T cells were present in HCC patients in at least a three log higher frequency compared to MAGE-A-specific CD8+ T cells. This holds true for both tested MAGE-A-specific epitopes, MAGE-A1_96_ and MAGE-A3_271_. The frequency of virus-specific CD8+ T cells, however, was not affected by the presence of HCC as similar frequencies were observed in HD (HCC: Median: 6.4*10^-3^; IQR: 7.1*10^-3^; HD: Median: 9.2*10^-3^; IQR: 1.2*10^-3^). Additionally, all virus-specific CD8+ T cells displayed an antigen-experienced phenotype in HCC patients and HD (Figure 3B). Thus, these results show that the low frequency and heterogeneous phenotype of MAGE-A-specific CD8+ T cells cannot be attributed to general tumor-specific factors that actively affect all antigen-specific CD8+ T cells but that this is rather a TAA-specific feature.

### Transarterial chemoembolization does not lead to stable induction or enhancement of circulating TAA-specific CD8+ T-cell responses

**Figure 4:**
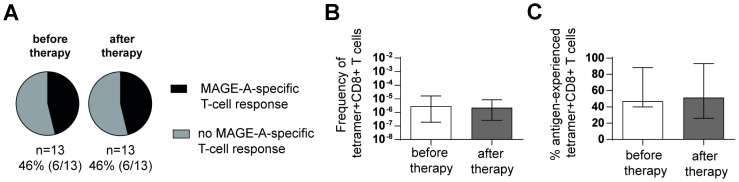
TACE does not enhance circulating TAA-specific CD8+ T-cell responses in HCC patients. Detection rates of MAGE-A-specific CD8+ T-cell responses (black versus grey: not detectable) before and after TACE are depicted in pie charts (**A**). Frequencies (**B**) and percentages of antigen-experienced (**C**) MAGE-A-specific CD8+ T-cell responses before and after TACE in HCC patients (HCC#4, 9, 19, 21–23, 27, 29, 32, 55–58) are displayed. Bar charts show median values with interquartile range. Statistical analysis was performed using binomial test (A) and non-parametric paired Wilcoxon test (B, C).

Previously, it was shown that ablative therapy leads to an activation and enhancement of TAA-specific CD8+ T cells [23]. Thus, we next analyzed the MAGE-A-specific CD8+ T-cell response in 13 patients of our cohort before and in average 3 months after transarterial chemoembolization (TACE) (Supplementary Table 1). As shown in Figure 4A, 6 out of 13 (46%) HCC patients displayed a MAGE-A-specific CD8+ T-cell response prior therapy and 6 out of 13 (46%) patients after TACE and therefore no increase in the circulating TAA-specific T-cell response was observed. In agreement with this finding, we also did not observe an increase in the overall frequency of MAGE-A-specific CD8+ T cells (Figure 4B) or in the percentage of antigen-experienced MAGE-A-specific CD8+ T cells (Figure 4C). Thus, these results indicate that TACE therapy has no lasting effect on the circulating TAA-specific CD8+ T-cell profile within our available sample collection.

### Circulating TAA-specific CD8+ T-cell responses are linked to the presence of TAA in tumor tissue

**Figure 5:**
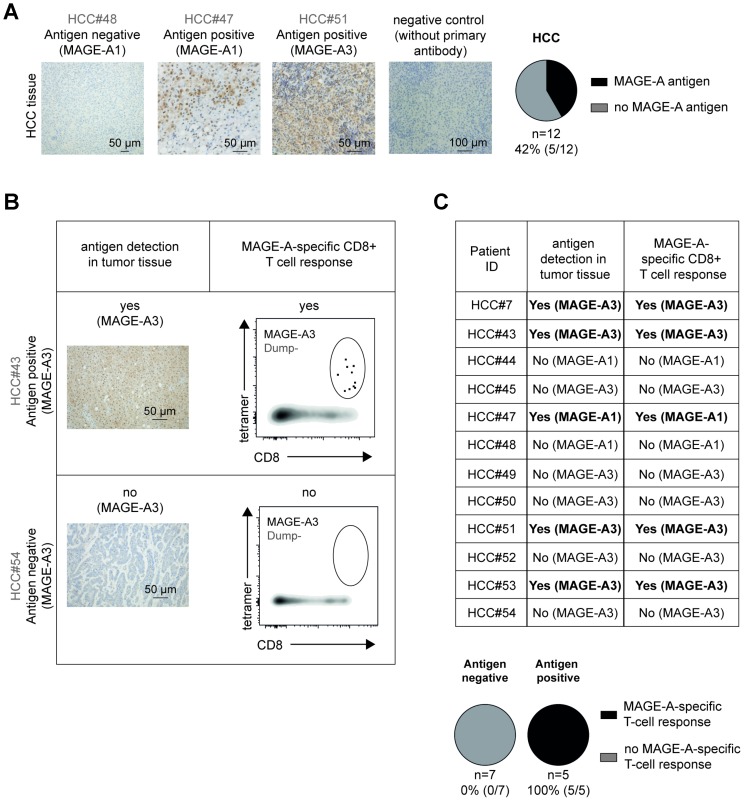
TAA-specific CD8+ T-cell responses in HCC patients are associated with the presence of TAA in tumor tissue. MAGE-A expression is heterogeneous in HCC tissue. Immunohistochemical analysis (MAGE-A: brown) of paraffin-embedded HCC tissue including negative controls (without primary antibody) and pie charts depicting the detection rate of MAGE-A (black: detectable versus grey: not detectable) are displayed (**A**). MAGE-A detection in HCC tissue and detection of circulating MAGE-A-specific CD8+ T cells correlates. Representative histological analyses (MAGE-A: brown), corresponding flow cytometry plots of circulating MAGE-A-specific CD8+ T-cell responses (**B**), tabular and pie chart analysis (**C**) are depicted.

One obvious explanation for the limited TAA-specific CD8+ T-cell response could be restricted antigen expression. To address this possibility, we analyzed available HCC tissue from 12 patients (Supplementary Table 1) for MAGE-A antigen expression by immunohistochemistry and included testis tissue as positive control (Supplementary Figure 2). As shown in Figure 5A and Supplementary Figure 3A, and in agreement with the literature [14], we found MAGE-A expression in 5 out of 12 tested samples (42%). Thereby, the level of antigen expression was quite heterogeneous as shown in the representative stainings in Figure 5A. Next, we compared the presence of antigen in tumor tissue with the detection of MAGE-A-specific CD8+ T-cell responses (Figure 5B and 5C). Importantly, in all HCC patients with detectable antigen, we also found MAGE-A-specific CD8+ T-cell responses. These MAGE-A-specific CD8+ T-cell populations were heterogeneously composed of phenotypically naïve and antigen-experienced cells (Supplementary Figure 3B). In contrast, none of the 7 patients without detectable antigen displayed a MAGE-A-specific CD8+ T-cell response. Thus, these results strongly suggest that presence of antigen is linked to the detection of circulating TAA-specific CD8+ T cells in HCC patients; however, still a decent proportion of these cells exhibit a naïve phenotype.

### Circulating TAA-specific CD8+ T cells in HCC patients do not display an exhausted phenotype

**Figure 6:**
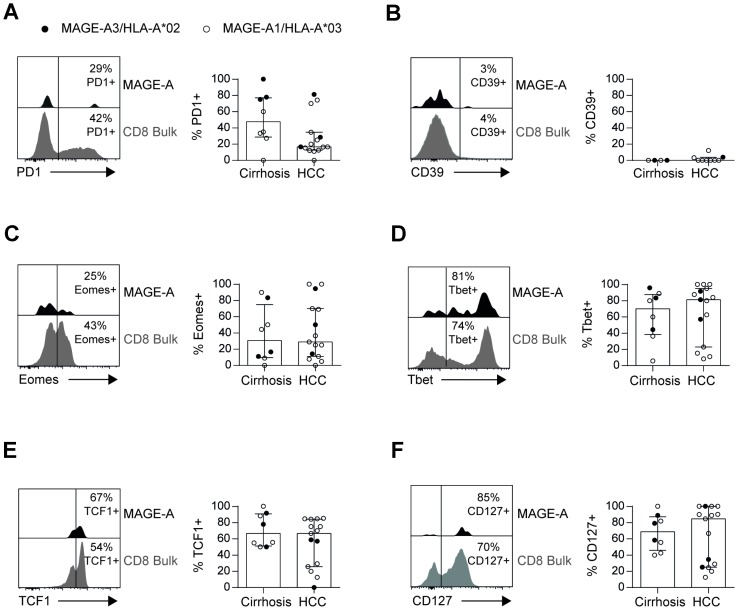
Circulating TAA-specific CD8+ T cells are not exhausted in HCC patients. Phenotypes of circulating MAGE-A-specific CD8+ T cells are similar in patients with liver cirrhosis and HCC patients with no signs of exhaustion. Representative flow cytometry plots and bar charts depicting expression of PD1 (**A**), CD39 (**B**), Eomes (**C**), Tbet (**D**), TCF1 (**E**) and CD127 (**F**) on antigen-experienced MAGE-A-specific CD8+ T cells are displayed. Statistical analysis was performed using unpaired *t*-test.

TAA-specific CD8+ T cells detectable in HCC patients have been reported to be dysfunctional and probably exhausted [6, 14]. To address this issue in more depth, we performed a comprehensive phenotypical analysis of the enriched antigen-experienced MAGE-A1_96_- and MAGE-A3_271_-specific CD8+ T cells obtained from blood of patients with HCC compared to liver cirrhosis, respectively. Surprisingly, as shown in Figure 6A, the majority of circulating antigen-experienced MAGE-A-specific CD8+ T cells did not express PD1 that is upregulated on exhausted cells. In line with this, we also did not observe a significant expression of the ectonucleotidase CD39 (Figure 6B) that has previously been reported to mark terminally exhausted CD8+ T-cell populations [26]. In addition, we also analyzed expression of the transcription factors Eomes and Tbet since high expression levels of Eomes accompanied by low Tbet expression has been linked to T-cell exhaustion [27]. However, MAGE-A-specific CD8+ T cells did not show strong Eomes expression in most patients (Figure 6C) contrasting an exhausted state of differentiation. In addition, the transcription factor Tbet was highly expressed in circulating MAGE-A-specific CD8+ T cells from patients suffering from liver cirrhosis and HCC (Figure 6D) rather pointing towards an effector/memory phenotype. In agreement with this finding, we found a high expression of the transcription factor TCF1 (Figure 6E) and the interleukin-7 receptor α-chain (CD127; Figure 6F) that are both central in the maintenance of memory T cells and that are typically downregulated in terminally exhausted CD8+ T cells. Taken together, these results demonstrate that circulating antigen-experienced MAGE-A-specific CD8+ T cells do not display the expected highly exhausted but rather an effector/memory phenotype. Noteworthy, we also did not find significant differences in the phenotype of MAGE-A-specific CD8+ T cells present in patients with liver cirrhosis versus HCC.

To address the question whether this surprising absence of an exhausted phenotype of circulating TAA-specific CD8+ T cells simply results from the lack of antigen expression and thus absence of potentially continuous antigen recognition, we phenotypically analyzed MAGE-A-specific CD8+ T cells in HCC patients with confirmed antigen expression in tumor tissue. We did not find any phenotypical differences of antigen-experienced MAGE-A-specific CD8+ T cells present in these HCC patients with verified antigen expression compared to the overall tested cohort of HCC patients in which we could not confirm respective TAA expression due to the lack of appropriate tumor samples (Supplementary Figure 3C–3G). This suggests that the absence of circulating TAA-specific CD8+ T-cell exhaustion cannot be explained by lack of antigen expression. Interestingly, however, immunohistochemical analyses of liver tissue from HCC patients revealed a rather low infiltration of the tumor by CD8+ T cells (Supplementary Figure 4). Thus, TAA-specific CD8+ T cells may not exhaust since antigen recognition is restricted by exclusion from the sites within the tissue where antigen is expressed.

## DISCUSSION

Our comprehensive study using a highly sensitive pMHCI-tetramer-based enrichment strategy gives important novel insights into circulating TAA-specific CD8+ T-cell responses in therapy-naïve HCC patients. First, in agreement with several previous reports, our results show that they are present only in a very low frequency. Indeed, we could not detect circulating TAA-specific CD8+ T-cell responses directly *ex vivo* but only after enrichment in the analyzed HLA-A*0201 or HLA-A*0301 positive patients. By this approach, NY-ESO-1 and Glypican-3-specific CD8+ T cells were still only detectable in less than 15% of patients, whereas MAGE-A-specific CD8+ T cells were present in about half of the patients. This is still a very low detection rate considering the high sensitivity of the enrichment strategy that allows e. g. the detection of HCV-specific CD8+ T cells in all patients tested [24]. The low detection rate despite pMHCI-tetramer enrichment indicates a very low frequency of circulating TAA-specific CD8+ T cells and may also explain their limited clinical efficacy in many tumor entities. Noteworthy, different hierarchies and immunodominances of TAA-specific CD8+ T-cell responses within different cohorts of HCC patients have been previously reported [6, 7, 14]. However, most of those were depending on *in vitro* expansion protocols prior ELISPOT assays that may not allow the full recapitulation of the TAA-specific CD8+ T-cell frequency *ex vivo*. These studies have also indicated a significant heterogeneity and no consistent hierarchy between different TAA-specific CD8+ T-cell responses within single cohorts of HCC patients [6, 7, 14]. Noteworthy, however, also by these approaches, the strongest responses were found to be directed against MAGE-A [6]. The significantly higher expression levels of MAGE-A versus NY-ESO-1 [8, 28] that have been reported not only in HCC but also in different tumors further support its better immunogenic potential and also fits to our finding with dominant MAGE-A-specific CD8+ T-cell responses.

Interestingly, however, the frequency of circulating MAGE-A-specific CD8+ T-cell responses was not higher in the analyzed HCC patients compared to patients with liver cirrhosis and healthy donors and was thus in the range of the naïve precursor frequency. This is a somewhat surprising finding since it suggests a limited TAA-specific CD8+ T-cell response induction and expansion in therapy-naïve HCC. This finding cannot be completely explained by absence of priming since a large fraction of TAA-specific CD8+ T cells displayed an antigen-experienced phenotype. However, within the same TAA-specific CD8+ T-cell responses present in HCC, T cells with a naïve phenotype could also be detected. This indicates an at least partially inefficient TAA-specific CD8+ T-cell priming and activation that may result in limited expansion and thus low frequencies hardly differing from naïve precursor frequencies. In contrast, virus-specific CD8+ T-cell responses present in the same patients showed a completely antigen-experienced phenotype, arguing against an actively ongoing general cancer-associated mechanism of improper T-cell priming in HCC. Combined, these results indicate an inefficient recruitment of circulating TAA-specific CD8+ T cells to the effector pool, the mechanisms of which are not well understood. One simple explanation could be the absence of respective antigens in the tumor tissue. However, we found antigen corresponding to TAA-specific CD8+ T cells expressed in 5 out of 12 analyzed tumor samples. In addition, the presence of antigen was associated with the detection of antigen-experienced TAA-specific CD8+ T cells, but a considerable proportion of cells with a naïve phenotype was still detectable. Thus, these results indicate that the inefficient activation and expansion of TAA-specific CD8+ T-cell responses cannot solely be explained by lack of antigen. It should be however noted that we cannot exclude the possibility that we may have a selection of escape mutations in the targeted TAA epitope in the analyzed patients.

It has been postulated that different HCC therapies such as radiofrequency ablation or TACE lead to an activation and enhancement of TAA-specific CD8+ T-cell responses probably reflecting activation by antigen release and/or induction of danger signals by tumor necrosis and or apoptosis [6, 23, 29–31]. However, in our study, by using a pMHCI-tetramer-based enrichment strategy, we did not find an increase in the detection rate, frequency or percentage of circulating antigen-experienced TAA-specific CD8+ T cells after TACE, further questioning the exclusive role of antigen in driving TAA-specific CD8+ T-cell activation. Importantly, we want to emphasize that blood sampling was conducted in average 28 days after TACE. Thus, the previously described transient increase in the frequencies of TAA-specific CD8+ T cells after therapy-induced local tumor destruction [7, 23, 30] was probably missed in our HLA-A2- and HLA-A3 restricted cohort. Of note, also a transient induction of TAA-specific T-cell responses after the local ablative TACE treatment could still have possible relevance for the course of combination therapies.

Probably the most important and surprising finding of our study is that detectable and circulating antigen-experienced TAA-specific CD8+ T cells in therapy-naïve HCC patients do not display a phenotype indicative of T-cell exhaustion. Indeed, the TAA-specific CD8+ T cells do not show high expression of PD1 or Eomes, both typical markers of T-cell exhaustion but rather strongly expressed TCF1 and CD127, markers characteristic for memory cells harboring a good proliferative capacity [32]. Of note, a moderate expression of inhibitory receptors on TAA-specific CD8+ T cells has also been previously reported in different cohorts of HCC patients with different underlying etiologies [14, 17, 18, 20]. Interestingly, these receptors were higher expressed on TAA-specific CD8+ T cells isolated from HCC tissue compared with T cells from tumor-free liver tissue or blood, although the tolerogenic liver environment itself has been shown to induce an upregulation of inhibitory receptors on T cells [18, 20]. Further analyses are therefore required to dissect the mechanisms underlying the expression of co-inhibitory receptors on intrahepatic TAA-specific CD8+ T cells. There are several possible explanations for our finding that at least peripheral TAA-specific CD8+ T cells do not show an exhausted phenotype due to ineffective activation. For example, failure of TAA presentation by antigen presenting cells as a consequence of decreased expression of HLA class I molecules or ineffective tumor antigen processing. This would also explain the overall low T-cell infiltration of the tumors in our study. In this context it is interesting to note that a strong MAGE-A-specific CD8+ T-cell response in concert with a significant T-cell infiltration has been reported in one patient where the tumor mass expanded out of the liver and infiltrated the peritoneum and diaphragmatic muscle [9]. Thus, these results link high antigen load and maybe even priming outside the liver with strong T-cell infiltration and TAA-specific CD8+ T-cell response. However, as such a scenario is rarely seen it further supports the hypothesis that absence of efficient priming is one hallmark of TAA-specific CD8+ T-cell failure in therapy-naïve HCC. It is also possible that other immune cell populations such as increases in regulatory T cells, myeloid-derived suppressor cells [17, 19, 21, 22] or tumor-associated macrophages [21] or lack of CD4+ T-cell help [18, 19] contribute to the observed TAA-specific CD8+ T-cell alterations.

The absence of TAA-specific CD8+ T-cell exhaustion in HCC is also surprising in light of a recent report showing that the PD1 inhibitor nivolumab leads to durable objective responses in patients with advanced HCC [5]. Also, recent gene expression analyses of HCC samples and deep single-cell RNA sequencing on T cells isolated from peripheral blood and HCCs revealed the existence of specific subsets of exhausted CD8+ T cells [33]. Indeed, 25% of HCCs were reported to have markers of an inflammatory response with high expression of PD1 that was also found to be highly expressed on HCC infiltrating T cells by single cell sequencing. While these results clearly show the existence of T-cell exhaustion in a subset of HCCs, they have not addressed the antigen-specificity of these responses. Based on our finding that circulating TAA-specific CD8+ T-cell responses are not exhausted, it is tempting to speculate that these T-cell responses are largely targeting neoantigens. Noteworthy, however, no correlation between the mutation load or the presence of neoantigens and the presence of inflammatory responses was observed in one study [34]. A similar lack of association has also been reported for other tumor entities with a similar rather modest mutational burden, such as prostate or ovarian cancer where the quality or clonality of neoantigens rather than the quantity has been suggested to shape the immune response [35, 36]. Clearly, further research is needed to define the targets of T-cell responses in HCC. However, our finding of not exhausted circulating TAA-specific CD8+ T-cell responses in a large cohort of HCC patients points towards the targeting of other antigens, such as neoantigens, that may drive T-cell exhaustion in natural HCC and may thus represent an optimal target of checkpoint inhibitor therapies.

Our study has some limitations. First, due to availability and technical feasibility, we could only apply a small selection of pMHCI-tetramers. Still, these pMHCI-tetramers detect TAA-specific CD8+ T cells that were shown to be immunodominant and to target antigens that were expressed in most tumors. Second, our study was limited to samples from the peripheral blood and not from tumor tissue since we did not have access to appropriate tissue samples. However, the peripheral blood represents the best accessible site for diagnostic measures and therapeutic interventions.

Taken together, the results of our comprehensive study based on a highly sensitive enrichment strategy gives novel important insights into HCC immunity by showing that circulating TAA-specific CD8+ T-cell responses are only present at very low frequencies, that they show a heterogeneous phenotype ranging from a naïve to an antigen-experienced phenotype indicating improper activation or priming, that they are not stably enhanced by TACE and probably most importantly, that they are not showing a phenotype indicative of T-cell exhaustion. These results show severe limitations of TAA-specific CD8+ T-cell responses present in therapy-naïve HCC that despite the presence of cognate antigen seem not be properly established. These results have important implications for immunotherapy as they highlight the challenge to induce TAA-specific CD8+ T-cell responses by vaccination or checkpoint inhibitors. They also suggest that other antigens, such as neoantigens, that have not been well-defined in HCC, may be primary targets of T-cell responses in therapy-naïve HCC. Clearly, important obstacles lie ahead that need to be addressed to fully uncover the potential of T-cell-targeted immunotherapy for the treatment of HCC.

## MATERIALS AND METHODS

### Study cohort

54 HCC patients (Supplementary Table 1), 28 healthy donors (Supplementary Table 2) and 29 patients with liver cirrhosis (Supplementary Table 3) were recruited at the Department of Medicine II of the University Hospital Freiburg, Germany. Written informed consent was obtained in all cases and the study was conducted according to the Declaration of Helsinki (1975), federal guidelines and local ethics committee regulations (Albert-Ludwigs-University, Freiburg, Germany, approvals 474/14 and 152/17). All analyzed HCC patients have not received immunotherapy and were mainly treated with local ablative TACE therapy (Supplementary Table 1).

### PBMC isolation

Peripheral blood mononuclear cells (PBMCs) were isolated from EDTA anti-coagulated blood by density-gradient centrifugation. For all analyses, frozen PBMCs were thawed in complete medium (RPMI 1640 with 10% fetal bovine serum, 1% penicillin/streptomycin and 1.5% 1M HEPES (all Thermo Fisher, Germany)) and incubated with 50 U/mL benzonase (Sigma, Germany) before further procedure.

### pMHCI-tetramers

pMHCI-tetramers consist of biotinylated pMHCI-monomers (provided by David A. Price) complexed by phycoerythrin (PE)- or allophycocyanin (APC)-labelled streptavidin at a molar ratio of 5:1. HLA-A*02-tetramers carrying previously described TAA epitopes (NY-ESO-1_157-165_: SLLMWITQA [12]; Glypican-3_521-530_: FLAELAYDL [18]; AFP_47-55_: ATIFFAQFV [6] and MAGE-A3_271-279_: FLWGPRALV [6]) or viral epitopes (Cytomegalovirus (CMV) pp65_495-503_: NLVPMVATV, Epstein-Barr virus (EBV) BMFL1_280-288_: GLCTLVAML and Influenza A virus M1_58-66_: GILGFVFTL); and HLA-A*03-tetramers carrying TAA epitopes (MAGE-A1_96_-_104_: SLFRAVITK [6] and Glypican-3_519-528_: QLRFLAELAY [6]) were used.

### pMHCI-tetramer-based enrichment

pMHCI-tetramer-based enrichment and subsequent calculation of TAA-specific CD8+ T-cell frequencies were performed as previously described by Alanio et al. [37]. Briefly, 50 Mio PBMCs were incubated for 30 min with PE- or APC-labeled pMHCI-tetramers. Anti-PE/APC MACS beads (Miltenyi Biotec, Germany) were used according to the manufacturer’s protocol.

$$\begin{array}{l} FrequencyofTetramer+CD8+T\text{​}cells\\ =\frac{\#Tetramer+CD8+Tcells(Enriched)}{absolutenumberof\text{​}CD8+T\text{​}\text{​}\text{​}cells} \end{array}$$

$$\begin{array}{l} absolutenumberofCD8+Tcells\\ =\frac{\#CD8+Tcells(PreEnrichment)x\#PBMCcount\;}{\#singlecells(PreEnrichment)} \end{array}$$

pMHCI-tetramers were titrated for optimal amount. The following tetramer amounts were used: 20 nM NY-ESO-1_157-165_; 3 nM Glypican-3_521-53,_ 3 nM, AFP_47-55,_ 3 nM, MAGE-A3_271-279,_ 1 nM MAGE-A1_96_-_104,_ 3 nM Glypican-3_519-528,_ 1 nM, CMV pp65_495-503,_ 1 nM, EBV BMFL1_280-288_ and 1 nM, Influenza A virus M1_58-66._

### Flow cytometry

For flow cytometry the following antibodies were used: anti-Eomes (WD1928), anti-Tbet (4B10), anti-CD14 (61D3), anti-CD19 (HIB19), anti-CCR7 (G043H7), anti-CD127 (A019D5), anti-CD45RA (HI100), anti-PD1 (EH12.2H7), anti-CD8 (HIT8a), Donkey anti-Rabbit IgG (Poly4064) (all BioLegend, San Diego, CA, USA); anti-TCF1 (C63D9) (Cell Signaling, Germany); anti-CD39 (TU66), and anti-CD8 (RPA-T8) (both BD Biosciences, Germany). Fixable Viability Dyes (eFluor780, eBioscience, Germany) was used for live/dead discrimination. FoxP3/Transcription Factor Staining Buffer Set (eBioscience, Germany) was applied according to the manufacturer’s instructions for intranuclear staining. Cells were fixed with paraformaldehyde (2% PFA) and analyzed using a LSRFortessa (BD Biosciences, Germany). Flow cytometry data were analyzed using FlowJo software V10 (Treestar, USA). Gating strategy is shown in Supplementary Figure 1. After exclusion of CD45RA+CCR7+ naïve T cells, marker analysis was restricted to patients showing at least five antigen-experienced MAGE-A-specific CD8+ T cells. Dump channel includes dead cells, CD14+ and CD19+ cells.

### HLA typing

HLA-typing was performed by next generation sequencing using commercially available primers (GenDx, Utrecht, The Netherlands) and run on a MiSeq system (Illumina). Data were analyzed using the NGSengine^®^ Software (GenDx).

### Immunohistochemistry

For MAGE-A1 and MAGE-A3 antigen detection in tumor tissue, paraffin-embedded slices from 12 HCC patients were analyzed. Sections were de-waxed in xylene/ethanol before target retrievel in citrate pH6 buffer (DakoCytomation Target Retrieval Solution Citrate pH6 (10×) (S2369) or DAKO Target Retrieval Solution, pH9 (10×) (S2367)) for 20 min. After peroxidase inactivation via Peroxidase Blocking (K5007: HRP EnVision FLEX Peroxidase Blocking Reagent (DAKO SM801), sections were stained with the specific primary anti- CD8 (C8/144B, 1:60, Dako), MAGE-A1 (MA454, 1:100) or MAGE-A3 (polyclonal, 1:100) (Sigma). EnVision FLEX mouse Linker (DAKO SM804) was used to enhance the MAGE staining. Antibodies were detected using a biotinylated anti-mouse IgG followed by an incubation with peroxidase-conjugated avidin. Sections were counterstained with Mayer´s hematoxylin (Sigma Aldrich, Germany).

### Statistics

Statistical analysis was performed with GraphPad Prism 6 (GraphPad Software, La Jolla, CA, USA).

## SUPPLEMENTARY MATERIALS





## Abbreviations

HCC: Hepatocellular carcinoma
TAA: tumor-associated antigen
AFP: a-fetoprotein
GPC-3: glypican-3
NY-ESO-1: New York esophageal squamous cell carcinoma
MAGE-A: melanoma-associated gene-A
pMHCI: peptide/MHCI
TACE: transarterial chemoembolization
HD: healthy donor

## References

[1] Balogh J, Victor D 3rd, Asham EH, Burroughs SG, Boktour M, Saharia A, Li X, Ghobrial RM, Monsour HP Jr Hepatocellular carcinoma: a review. J Hepatocell Carcinoma. 2016; 3:41–53. 10.2147/JHC.S61146. 27785449 PMC5063561

[2] Foerster F, Hess M, Gerhold-Ay A, Marquardt JU, Becker D, Galle PR, Schuppan D, Binder H, Bockamp E The immune contexture of hepatocellular carcinoma predicts clinical outcome. Sci Rep. 2018; 8:5351. 10.1038/s41598-018-21937-2. 29599491 PMC5876395

[3] Yao W, He JC, Yang Y, Wang JM, Qian YW, Yang T, Ji L The Prognostic Value of Tumor-infiltrating Lymphocytes in Hepatocellular Carcinoma: a Systematic Review and Meta-analysis. Sci Rep. 2017; 7:7525. 10.1038/s41598-017-08128-1. 28790445 PMC5548736

[4] Gabrielson A, Wu Y, Wang H, Jiang J, Kallakury B, Gatalica Z, Reddy S, Kleiner D, Fishbein T, Johnson L, Island E, Satoskar R, Banovac F, et al Intratumoral CD3 and CD8 T-cell Densities Associated with Relapse-Free Survival in HCC. Cancer Immunol Res. 2016; 4:419–430. 10.1158/2326-6066.CIR-15-0110. 26968206 PMC5303359

[5] El-Khoueiry AB, Sangro B, Yau T, Crocenzi TS, Kudo M, Hsu C, Kim TY, Choo SP, Trojan J, Welling TH 3rd, Meyer T, Kang YK, Yeo W, et al Nivolumab in patients with advanced hepatocellular carcinoma (CheckMate 040): an open-label, non-comparative, phase 1/2 dose escalation and expansion trial. Lancet. 2017; 389:2492–2502. 10.1016/S0140-6736(17)31046-2. 28434648 PMC7539326

[6] Flecken T, Schmidt N, Hild S, Gostick E, Drognitz O, Zeiser R, Schemmer P, Bruns H, Eiermann T, Price DA, Blum HE, Neumann-Haefelin C, Thimme R Immunodominance and functional alterations of tumor-associated antigen-specific CD8+ T-cell responses in hepatocellular carcinoma. Hepatology. 2014; 59:1415–1426. 10.1002/hep.26731. 24002931 PMC4139003

[7] Mizukoshi E, Nakamoto Y, Arai K, Yamashita T, Sakai A, Sakai Y, Kagaya T, Yamashita T, Honda M, Kaneko S Comparative analysis of various tumor-associated antigen-specific t-cell responses in patients with hepatocellular carcinoma. Hepatology. 2011; 53:1206–1216. 10.1002/hep.24149. 21480325

[8] Sideras K, Bots SJ, Biermann K, Sprengers D, Polak WG, IJzermans JN, de Man RA, Pan Q, Sleijfer S, Bruno MJ, Kwekkeboom J Tumour antigen expression in hepatocellular carcinoma in a low-endemic western area. Br J Cancer. 2015; 112:1911–1920. 10.1038/bjc.2015.92. 26057582 PMC4580401

[9] Zerbini A, Pilli M, Soliani P, Ziegler S, Pelosi G, Orlandini A, Cavallo C, Uggeri J, Scandroglio R, Crafa P, Spagnoli GC, Ferrari C, Missale G *Ex vivo* characterization of tumor-derived melanoma antigen encoding gene-specific CD8+cells in patients with hepatocellular carcinoma . J Hepatol. 2004; 40:102–109. 10.1016/S0168-8278(03)00484-7. 14672620

[10] Butterfield LH, Ribas A, Potter DM, Economou JS Spontaneous and vaccine induced AFP-specific T cell phenotypes in subjects with AFP-positive hepatocellular cancer. Cancer Immunol Immunother. 2007; 56:1931–1943. 10.1007/s00262-007-0337-9. 17522860 PMC11030770

[11] Komori H, Nakatsura T, Senju S, Yoshitake Y, Motomura Y, Ikuta Y, Fukuma D, Yokomine K, Harao M, Beppu T, Matsui M, Torigoe T, Sato N, et al Identification of HLA-A2- or HLA-A24-restricted CTL epitopes possibly useful for glypican-3-specific immunotherapy of hepatocellular carcinoma. Clin Cancer Res. 2006; 12:2689–2697. 10.1158/1078-0432.CCR-05-2267. 16675560

[12] Korangy F, Ormandy LA, Bleck JS, Klempnauer J, Wilkens L, Manns MP, Greten TF Spontaneous tumor-specific humoral and cellular immune responses to NY-ESO-1 in hepatocellular carcinoma. Clin Cancer Res. 2004; 10:4332–4341. 10.1158/1078-0432.CCR-04-0181. 15240519

[13] Thimme R, Neagu M, Boettler T, Neumann-Haefelin C, Kersting N, Geissler M, Makowiec F, Obermaier R, Hopt UT, Blum HE, Spangenberg HC Comprehensive analysis of the alpha-fetoprotein-specific CD8+ T cell responses in patients with hepatocellular carcinoma. Hepatology. 2008; 48:1821–1833. 10.1002/hep.22535. 19003875

[14] Gehring AJ, Ho ZZ, Tan AT, Aung MO, Lee KH, Tan KC, Lim SG, Bertoletti A Profile of tumor antigen-specific CD8 T cells in patients with hepatitis B virus-related hepatocellular carcinoma. Gastroenterology. 2009; 137:682–690. 10.1053/j.gastro.2009.04.045. 19394336

[15] Chen CH, Chen GJ, Lee HS, Huang GT, Yang PM, Tsai LJ, Chen DS, Sheu JC Expressions of cancer-testis antigens in human hepatocellular carcinomas. Cancer Lett. 2001; 164:189–195. 10.1016/S0304-3835(01)00379-2. 11179834

[16] Tahara K, Mori M, Sadanaga N, Sakamoto Y, Kitano S, Makuuchi M Expression of the MAGE Gene Family in Human Hepatocellular Carcinoma. American Cancer Society. 1999; 85:1234–1240. 10.1002/(SICI)1097-0142(19990315)85:6&3c1234::AID-CNCR4&3e3.0.CO;2-7. 10189127

[17] Inada Y, Mizukoshi E, Seike T, Tamai T, Iida N, Kitahara M, Yamashita T, Arai K, Terashima T, Fushimi K, Yamashita T, Honda M, Kaneko S Characteristics of Immune Response to Tumor-Associated Antigens and Immune Cell Profile in Patients With Hepatocellular Carcinoma. Hepatology. 2019; 69:653–65. 10.1002/hep.30212. 30102778

[18] Zhou G, Sprengers D, Boor PP, Doukas M, Schutz H, Mancham S, Pedroza-Gonzalez A, Polak WG, de Jonge J, Gaspersz M, Dong H, Thielemans K, Pan Q, et al Antibodies Against Immune Checkpoint Molecules Restore Functions of Tumor-Infiltrating T Cells in Hepatocellular Carcinomas. Gastroenterology. 2017; 153:1107–1119.e10. 10.1053/j.gastro.2017.06.017. 28648905

[19] Breous E, Thimme R Potential of immunotherapy for hepatocellular carcinoma. J Hepatol. 2011; 54:830–834. 10.1016/j.jhep.2010.10.013. 21145836

[20] Kim HD, Song GW, Park S, Jung MK, Kim MH, Kang HJ, Yoo C, Yi K, Kim KH, Eo S, Moon DB, Hong SM, Ju YS, et al Association Between Expression Level of PD1 by Tumor-Infiltrating CD8(+) T Cells and Features of Hepatocellular Carcinoma. Gastroenterology. 2018; 155:1936–1950.e17. 10.1053/j.gastro.2018.08.030. 30145359

[21] Mizukoshi E, Kaneko S Antigen-specific T cell responses in hepatocellular carcinoma Immunotherapy of Hepatocellular Carcinoma Springer International Publishing AG 2017:39–50. 10.1007/978-3-319-64958-0_3.

[22] Greten TF, Duffy AG, Korangy F Hepatocellular carcinoma from an immunologic perspective. Clin Cancer Res. 2013; 19:6678–6685. 10.1158/1078-0432.CCR-13-1721. 24030702 PMC3867536

[23] Mizukoshi E, Yamashita T, Arai K, Sunagozaka H, Ueda T, Arihara F, Kagaya T, Yamashita T, Fushimi K, Kaneko S Enhancement of tumor-associated antigen-specific T cell responses by radiofrequency ablation of hepatocellular carcinoma. Hepatology. 2013; 57:1448–1457. 10.1002/hep.26153. 23174905

[24] Nitschke K, Flecken T, Schmidt J, Gostick E, Marget M, Neumann-Haefelin C, Blum HE, Price DA, Thimme R Tetramer enrichment reveals the presence of phenotypically diverse hepatitis C virus-specific CD8+ T cells in chronic infection. J Virol. 2015; 89:25–34. 10.1128/JVI.02242-14. 25320295 PMC4301109

[25] Wieland D, Kemming J, Schuch A, Emmerich F, Knolle P, Neumann-Haefelin C, Held W, Zehn D, Hofmann M, Thimme R TCF1(+) hepatitis C virus-specific CD8(+) T cells are maintained after cessation of chronic antigen stimulation. Nat Commun. 2017; 8:15050. 10.1038/ncomms15050. 28466857 PMC5418623

[26] Gupta PK, Godec J, Wolski D, Adland E, Yates K, Pauken KE, Cosgrove C, Ledderose C, Junger WG, Robson SC, Wherry EJ, Alter G, Goulder PJ, et al CD39 Expression Identifies Terminally Exhausted CD8+ T Cells. PLoS Pathog. 2015; 11:e1005177. 10.1371/journal.ppat.1005177. 26485519 PMC4618999

[27] Paley MA, Kroy DC, Odorizzi PM, Johnnidis JB, Dolfi DV, Barnett BE, Bikoff EK, Robertson EJ, Lauer GM, Reiner SL, Wherry EJ Progenitor and terminal subsets of CD8+ T cells cooperate to contain chronic viral infection. Science. 2012; 338:1220–1225. 10.1126/science.1229620. 23197535 PMC3653769

[28] Kerkar SP, Wang ZF, Lasota J, Park T, Patel K, Groh E, Rosenberg SA, Miettinen MM MAGE-A is More Highly Expressed Than NY-ESO-1 in a Systematic Immunohistochemical Analysis of 3668 Cases. J Immunother. 2016; 39:181–187. 10.1097/CJI.0000000000000119. 27070449 PMC4831141

[29] Zerbini A, Pilli M, Penna A, Pelosi G, Schianchi C, Molinari A, Schivazappa S, Zibera C, Fagnoni FF, Ferrari C, Missale G Radiofrequency thermal ablation of hepatocellular carcinoma liver nodules can activate and enhance tumor-specific T-cell responses. Cancer Res. 2006; 66:1139–1146. 10.1158/0008-5472.CAN-05-2244. 16424051

[30] Mizukoshi E, Nakamoto Y, Arai K, Yamashita T, Mukaida N, Matsushima K, Matsui O, Kaneko S Enhancement of tumor-specific T-cell responses by transcatheter arterial embolization with dendritic cell infusion for hepatocellular carcinoma. Int J Cancer. 2010; 126:2164–2174. 10.1002/ijc.24882. 19739081

[31] Ayaru L, Pereira SP, Alisa A, Pathan AA, Williams R, Davidson B, Burroughs AK, Meyer T, Behboudi S Unmasking of alpha-fetoprotein-specific CD4(+) T cell responses in hepatocellular carcinoma patients undergoing embolization. J Immunol. 2007; 178:1914–1922. 10.4049/jimmunol.178.3.1914. 17237442

[32] Jeannet G, Boudousquie C, Gardiol N, Kang J, Huelsken J, Held W Essential role of the Wnt pathway effector Tcf-1 for the establishment of functional CD8 T cell memory. Proc Natl Acad Sci USA. 2010; 107:9777–9782. 10.1073/pnas.0914127107. 20457902 PMC2906901

[33] Zheng C, Zheng L, Yoo JK, Guo H, Zhang Y, Guo X, Kang B, Hu R, Huang JY, Zhang Q, Liu Z, Dong M, Hu X, et al Landscape of Infiltrating T Cells in Liver Cancer Revealed by Single-Cell Sequencing. Cell. 2017; 169:1342–1356. 28622514 10.1016/j.cell.2017.05.035

[34] Sia D, Jiao Y, Martinez-Quetglas I, Kuchuk O, Villacorta-Martin C, Castro de Moura M, Putra J, Camprecios G, Bassaganyas L, Akers N, Losic B, Waxman S, Thung SN, et al Identification of an Immune-specific Class of Hepatocellular Carcinoma, Based on Molecular Features. Gastroenterology. 2017; 153:812–826. 10.1053/j.gastro.2017.06.007. 28624577 PMC12166766

[35] Balachandran VP, Luksza M, Zhao JN, Makarov V, Moral JA, Remark R, Herbst B, Askan G, Bhanot U, Senbabaoglu Y, Wells DK, Cary CIO, Grbovic-Huezo O, et al Identification of unique neoantigen qualities in long-term survivors of pancreatic cancer. Nature. 2017; 551:512–516. 10.1038/nature24462. 29132146 PMC6145146

[36] Luksza M, Riaz N, Makarov V, Balachandran VP, Hellmann MD, Solovyov A, Rizvi NA, Merghoub T, Levine AJ, Chan TA, Wolchok JD, Greenbaum BD A neoantigen fitness model predicts tumour response to checkpoint blockade immunotherapy. Nature. 2017; 551:517–520. 10.1038/nature24473. 29132144 PMC6137806

[37] Alanio C, Lemaitre F, Law HK, Hasan M, Albert ML Enumeration of human antigen-specific naive CD8+ T cells reveals conserved precursor frequencies. Blood. 2010; 115:3718–3725. 10.1182/blood-2009-10-251124. 20200354

