# Supplementary material for: Inefficient induction of circulating TAA-specific CD8+ T-cell responses in hepatocellular carcinoma
Source: Oncotarget. 2019 Aug 27;10(50):5194–206. doi: 10.18632/oncotarget.27146 (PMC6718268; doi:10.18632/oncotarget.27146)
Supplement: Supplementary file 1 [file oncotarget-10-5194-s001.pdf]

Inefficient induction of circulating TAA-specific CD8+ T-cell responses in hepatocellular carcinoma

SUPPLEMENTARY MATERIALS

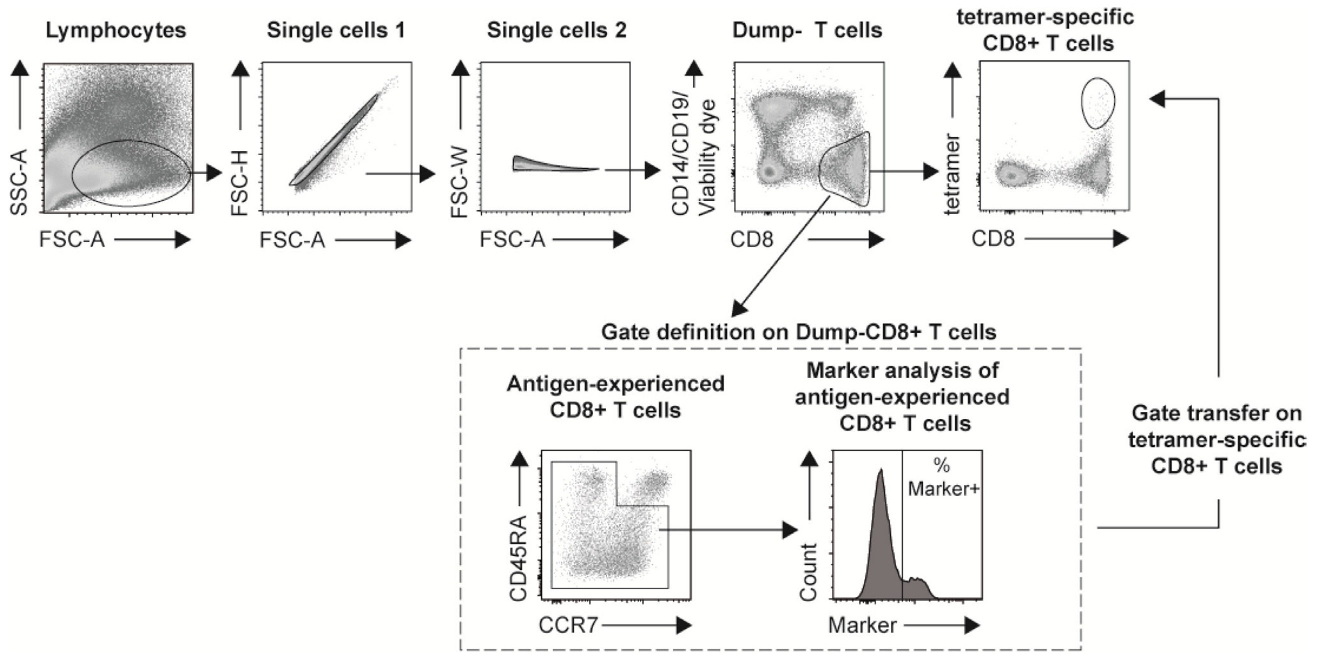

Supplementary Figure 1: Gating strategy for phenotypic analysis of TAA-specific CD8+ T cells. Dump channel includes dead cells, CD14+ and CD19+ cells.

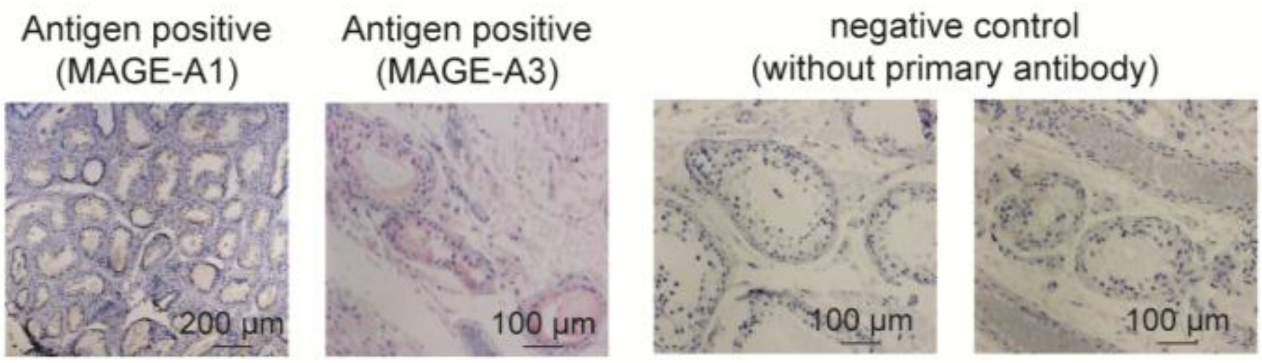

Supplementary Figure 2: Histochemical control analysis of paraffin-embedded testis tissue. Immunohistochemical staining of paraffin-embedded testis tissue samples. Antigen positive staining of testis tissue for MAGE-A (MAGE-A1: brown, MAGE-A3: red) including and negative control (without primary antibody) are shown.

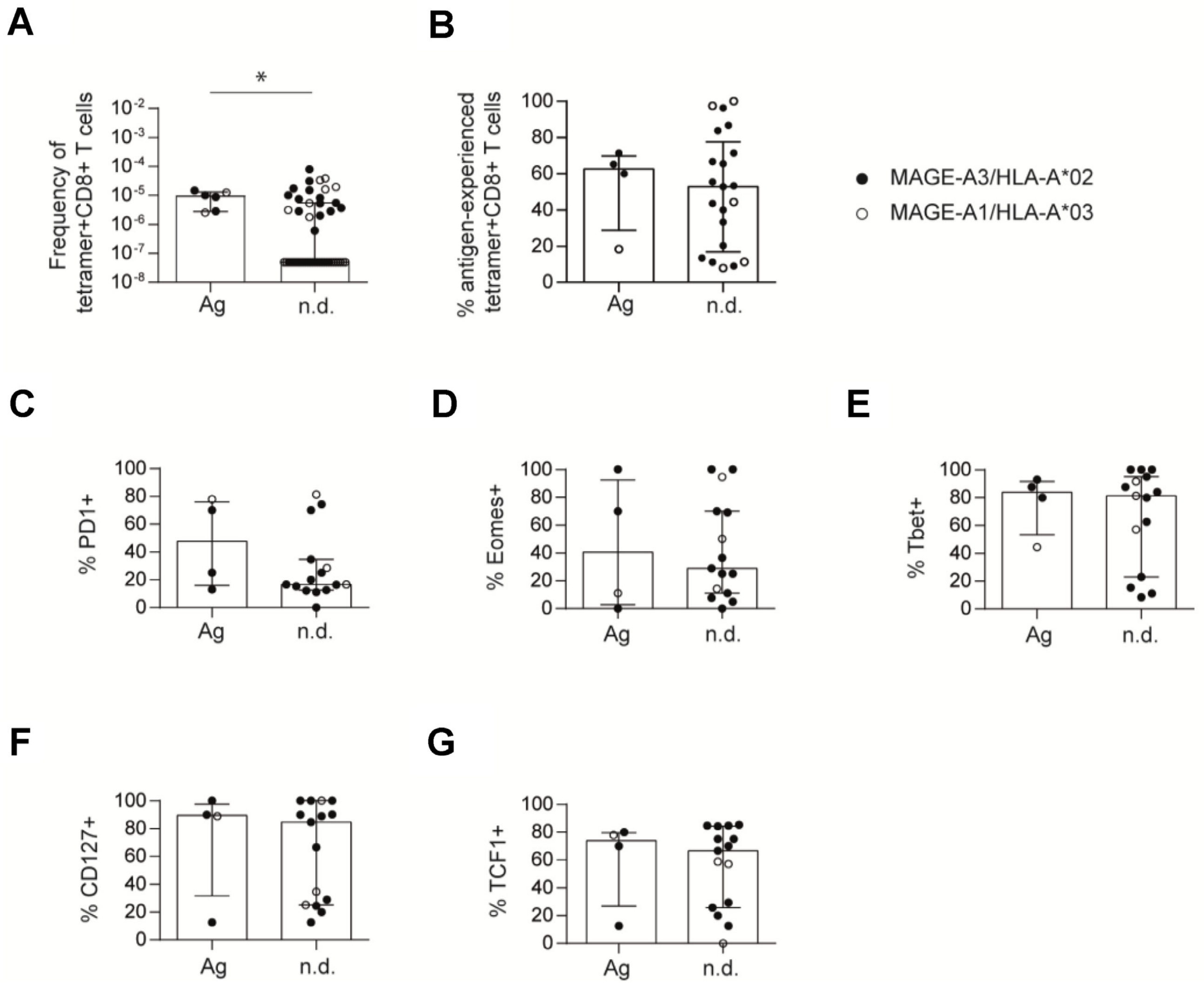

Supplementary Figure 3: Circulating TAA-specific CD8+ T cells are not exhausted in HCC despite antigen expression. Frequencies (**A**) and percentages of detectable antigen-experienced (**B**) circulating MAGE-A-specific CD8+ T cells in HCC patients with verified antigen expression in tumor tissue (Ag) and in HCC patients in whom antigen expression was not determined (n.d.) are depicted as bar charts. Bar charts depicting percentages of PD1+ (**C**), Eomes+ (**D**), Tbet+ (**E**), CD127+ (**F**) and TCF1+ (**G**) antigen-experienced MAGE-A-specific CD8+ T cells in HCC patients with verified antigen expression in tumor tissue (Ag) and in HCC patients in which antigen expression was not determined (n.d.) are displayed. Statistical analysis was performed via non-parametric Mann-Whitney test ($^{*}p < 0.005$).

A

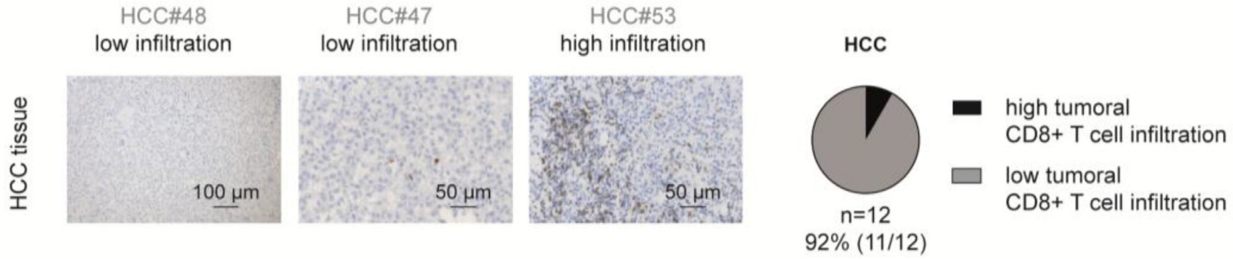

B

Patient ID	antigen detection in tumor tissue	CD8+ T-cell infiltration	median CD8+ T-cell count/mm2
HCC#7	**Yes (MAGE-A3)**	Low	3
HCC#43	**Yes (MAGE-A3)**	Low	0
HCC#44	No (MAGE-A1)	Low	2.5
HCC#45	No (MAGE-A3)	Low	0
HCC#47	**Yes (MAGE-A1)**	Low	5
HCC#48	No (MAGE-A1)	Low	2
HCC#49	No (MAGE-A3)	Low	1
HCC#50	No (MAGE-A3)	Low	0
HCC#51	**Yes (MAGE-A3)**	High	15.5
HCC#52	No (MAGE-A3)	Low	0
HCC#53	**Yes (MAGE-A3)**	Low	2
HCC#54	No (MAGE-A3)	Low	3.5

Supplementary Figure 4: Low CD8+ T-cell infiltration into HCC tissue. Immunohistochemical staining (CD8+ T cells: brown) of paraffin-embedded HCC tissue and a pie chart depicting qualitative CD8+ T-cell infiltration rates (**A**; high: black, counts of at least 15 CD8+ T cells within the tumor; low: grey, only scattered CD8+ T cells within the tumor) and tabular analysis (**B**) are displayed. Depicted are the median values of CD8+ T cells/mm^2.

Supplementary Table 1: Study cohort of HCC patients. See Supplementary Table 1

Supplementary Table 2: Study cohort of healthy donors

Patient ID	Age [years]	Sex	HLA type	CD8+ T-cell response						
				HLA-A2 MAGE-A3$_{271-279}$	HLA-A2 NY-ESO-1$_{157-165}$	HLA-A2 CMV-pp65$_{495-503}$	HLA-A2 EBV BMLF1$_{280-288}$	HLA-A2 Influenza M1$_{58-66}$	HHLA-A3 MAGE-A1$_{96-104}$	HLA-A3 Glypican-351$_{9-528}$
HD#1	52	M	A0201	■	■					
HD#2	59	F	A0201	■	■					
HD#3	64	M	A0201	■	■	■				
HD#4	59	M	A0201 and A0301	□	□				■	
HD#5	53	M	A0201 and A0301	□	□				■	
HD#6	79	F	A0201	■	■					
HD#7	58	F	A0201	■	■					
HD#8	57	M	A0201	■	■					
HD#9	54	F	A0201	■	■					
HD#10	56	F	A0201	■	■					
HD#11	88	M	A0201	■	■					
HD#12	54	F	A0201	■	■					
HD#13	56	F	A0201	■	■					
HD#14	57	F	A0201	■	□					
HD#15	52	F	A0201	■	■					
HD#16	55	F	A0201	■	■					
HD#17	54	F	A0201 and A0301	■	■				■	
HD#18	64	M	A0301						■	
HD#19	65	F	A0301	■	■					
HD#20	59	M	A0301						■	
HD#21	52	M	A0301	■	■					
HD#22	63	M	A0201 and A0301	■	■				□	
HD#23	58	M	A0201	■	■					
HD#24	74	M	A0201 and A0301						■	□
HD#25	36	M	A0201 and A0301	■	■		■		■	□
HD#26	47	F	A0201	■	■		■			
HD#27	42	M	A0301						■	
HD#28	36	M	A03001						■	□

Abbreviations: M: Male, F: Female, Patients with other HLA types than HLA-A*0201 and/or HLA-A*0301 are excluded. ■ tested positive; □ tested negative..

Supplementary Table 3: Study cohort of patients with liver cirrhosis

Patient ID	Age [years]	Sex	Etiology	Child score	HLA type	CD8+ T-cell response						
						HLA-A2 MAGE-A3$_{271-279}$	HLA-A2 NY-ESO-1$_{157-165}$	HLA-A2 CMV-pp65$_{495-503}$	HLA-A2 EBV BMLF1$_{280-288}$	HLA-A2 Influenza M1$_{58-66}$	HLA-A3 MAGE-A1$_{96-104}$	HLA-A3 Glypican-3$_{519-528}$
Cirrhosis#1	57	M	HCV	A	A0301						■	□
Cirrhosis#2	57	M	HCV	A	A0301						□	
Cirrhosis#3	57	F	HBV	A	A0201	■	□					
Cirrhosis#4	53	M	NASH	A	A0301						□	□
Cirrhosis#5	56	M	NASH	A	A0201	□	□		■	■		
Cirrhosis#6	51	M	HCV	C	A0201	□	□		■			
Cirrhosis#7	64	M	NASH	A	A0201	□	□					
Cirrhosis#8	64	F	HCV	A	A0301						■	
Cirrhosis#9	77	M	ASH	B	A0301						□	
Cirrhosis#10	50	M	Other	B	A0301						□	
Cirrhosis#11	55	F	ASH	A	A0201	■	□					
Cirrhosis#12	64	M	Other	A	A0201	■	□					
Cirrhosis#13	64	F	ASH	A	A0201	■						
Cirrhosis#14	49	M	HCV	B	A0201	■	□					
Cirrhosis#15	55	F	NASH	A	A0201	□	□					
Cirrhosis#16	78	M	ASH	A	A0301						■	
Cirrhosis#17	63	M	ASH	A	A0201 and A0301	□	■				□	
Cirrhosis#18	76	F	ASH	B	A0301						□	
Cirrhosis#19	55	F	Other	A	A0301						■	
Cirrhosis#20	57	M	ASH	B	A0201 and A0301	□	□				■	□
Cirrhosis#21	57	F	ASH	B	A0301						■	
Cirrhosis#22	61	M	ASH	B	A0201 and A0301						□	
Cirrhosis#23	72	M	ASH	B	A0201	□	□					
Cirrhosis#24	66	M	ASH	B	A0301						■	
Cirrhosis#25	50	F	ASH	B	A0201	□	□					
Cirrhosis#26	71	F	ASH	C	A0301						■	
Cirrhosis#27	56	F	ASH	A	A0201	■	□		■	■		
Cirrhosis#28	71	M	ASH	B	A0201	■						
Cirrhosis#29	51	M	NASH	A	A0201	■						

Abbreviations: M: Male, F: Female, HCV: Hepatitis C virus, HBV: hepatitis B virus, ASH: alcohol induced steatohepatitis, NASH: non-alcoholic steatohepatis, Other: combinations of risk factors or haemochromatosis. Patients with other HLA types than HLA-A*0201 and/or HLA-A*0301 are excluded. ■ tested positive; □ tested negative
