# Supplementary material for: Inefficient induction of circulating TAA-specific CD8+ T-cell responses in hepatocellular carcinoma
Source: Oncotarget. 2019 Aug 27;10(50):5194–206. doi: 10.18632/oncotarget.27146 (PMC6718268; doi:10.18632/oncotarget.27146)
Supplement: Supplementary file 2 [file oncotarget-10-5194-s002.docx]

## **Supplementary Table 1:** Study cohort of HCC patients.

Abbreviations: M: Male, F: Female, HCV: Hepatitis C virus, HBV: hepatitis B virus, ASH: alcohol induced steatohepatitis, NASH: non-alcoholic steatohepatitis, Other: combinations of risk factors or haemochromatosis. Patients with other HLA types than HLA-A*0201 and/or HLA-A*0301 are excluded. ■ tested positive; □ tested negative n.d.: not determined, pos.: positive. Median timespan of blood collection after TACE: 28 days (2days-6 months).

| Patient ID | Age [years] | Sex | Etiology | Child score | BCLC score | HLA- type | Treatment prior inclusion | CD8+ T-cell response | | | | | | | Phenotypic analysis |
| --- | --- | --- | --- | --- | --- | --- | --- | --- | --- | --- | --- | --- | --- | --- | --- |
|  |  |  |  |  |  |  |  | HLA-A2  MAGE-A3_271-279_ | HLA-A2  NY-ESO-1_157-165_ | HLA-A2  CMV-pp65_495-503_ | HLA-A2  EBV BMLF1_280-288_ | HLA-A2 Influenza M1_58-66_ | HLA-A3  MAGE-A1_96-104_ | HLA-A3  Glypican-3_519-528_ |  |
| HCC#1 | 66 | M | ASH | C | D | A0201 | No | □ | □ |  |  |  |  |  | Therapy-naive |
| HCC#2 | 52 | M | ASH | A | A | A0201 | No | □ | □ |  |  |  |  |  | Therapy-naive |
| HCC#3 | 52 | F | HCV | A | A | A0301 | No |  |  |  |  |  | ■ | □ | Therapy-naive |
| HCC#4 | 77 | F | HCV | A | A | A0301 | No |  |  |  |  |  | ■ | □ | Therapy-naive (before TACE) and  3 months after TACE |
| HCC#5 | 74 | M | HBV | A | C | A0201 | No | ■ | ■ |  |  |  |  |  | Therapy-naive |
| HCC#6 | 59 | M | ASH | C | A | A0201 | No | □ | □ |  |  |  |  |  | Therapy-naive |
| HCC#7 | 60 | F | HBV | A | 0 | A0201 | No | ■ | □ |  |  |  |  |  | Therapy-naive |
| HCC#8 | 56 | M | Other | no | B | A0301 | No |  |  |  |  |  | ■ | □ | Therapy-naive with corresponding antigen expression |
| HCC#9 | 78 | M | Other | A | B | A0201 | No | ■ | ■ |  | ■ |  |  |  | Therapy-naive (before TACE) and  6 months after TACE |
| HCC#10 | 75 | M | ASH | C | C | A0201 | No | □ | □ |  |  |  |  |  | Therapy-naive |
| HCC#11 | 54 | M | HCV | A | C | A0201 and A0301 | No |  |  |  |  |  | ■ | □ | Therapy-naive |
| HCC#12 | 63 | M | NASH | A | A | A0301 | No |  |  |  |  |  | ■ | □ | Therapy-naive |
| HCC#13 | 75 | M | ASH | A | B | A0301 | No |  |  |  |  |  | □ | □ | Therapy-naive |

| Patient ID | Age [years] | Sex | Etiology | Child score | BCLC score | HLA- type | Treatment prior inclusion | CD8+ T-cell response | | | | | | | Phenotypic analysis |
| --- | --- | --- | --- | --- | --- | --- | --- | --- | --- | --- | --- | --- | --- | --- | --- |
|  |  |  |  |  |  |  |  | HLA-A2  MAGE-A3_271-279_ | HLA-A2  NY-ESO-1_157-165_ | HLA-A2  CMV-pp65_495-503_ | HLA-A2  EBV BMLF1_280-288_ | HLA-A2 Influenza M1_58-66_ | HLA-A3  MAGE-A1_96-104_ | HLA-A3  Glypican-3_519-528_ |  |
| HCC#14 | 77 | M | Other | A | B | A0301 | No |  |  |  |  |  | ■ | □ | Therapy-naive |
| HCC#15 | 80 | M | NASH | A | A | A0201 | No | □ | □ |  |  |  |  |  | Therapy-naive |
| HCC#16 | 61 | M | ASH | A | A | A0201 | No | ■ | □ |  |  |  |  |  | Therapy-naive |
| HCC#17 | 82 | M | ASH | A | A | A0201 | No | ■ | □ |  |  |  |  |  | Therapy-naive |
| HCC#18 | 58 | M | ASH | B | A | A0201 | No | ■ | □ |  |  |  |  |  | Therapy-naive |
| HCC#19 | 81 | F | n.d. | A | A | A0301 | No |  |  |  |  |  | □ | □ | Therapy-naive (before TACE) and after 3 months after TACE |
| HCC#20 | 63 | M | ASH | A | B | A0201 | No | □ | □ |  |  |  |  |  | Therapy-naiv |
| HCC#21 | 76 | M | ASH | A | C | A0301 | No |  |  |  |  |  | □ | □ | Therapy-naive (before TACE) and 3 months after TACE |
| HCC#22 | 70 | M | ASH | B | B | A0201 | No | ■ | □ |  |  |  |  |  | Therapy-naive (before TACE) and 2 days after TACE |
| HCC#23 | 62 | M | ASH | A | B | A0201 | No | ■ | ■ | ■ |  |  |  |  | Therapy-naive (before TACE) and 2 days after TACE |
| HCC#24 | 59 | M | Other | A | C | A0201 and A0301 | No | □ | □ |  |  |  |  |  | Therapy-naive |
| HCC#25 | 65 | M | ASH | B | B | A0201 | No | □ | ■ | ■ |  | ■ |  |  | Therapy-naive |
| HCC#26 | 58 | F | Other | B | A | A0201 | No | □ | □ |  | ■ | □ |  |  | Therapy-naive |

| Patient ID | Age [years] | Sex | Etiology | Child score | BCLC score | HLA- type | Treatment prior inclusion | CD8+ T-cell response | | | | | | | Phenotypic analysis |
| --- | --- | --- | --- | --- | --- | --- | --- | --- | --- | --- | --- | --- | --- | --- | --- |
|  |  |  |  |  |  |  |  | HLA-A2  MAGE-A3_271-279_ | HLA-A2  NY-ESO-1_157-165_ | HLA-A2  CMV-pp65_495-503_ | HLA-A2  EBV BMLF1_280-288_ | HLA-A2 Influenza M1_58-66_ | HLA-A3  MAGE-A1_96-104_ | HLA-A3  Glypican-3_519-528_ |  |
| HCC#27 | 80 | M | ASH | A | B | A0301 | No |  |  |  |  |  | ■ | □ | Therapy-naive (before TACE) and 2 days after TACE |
| HCC#28 | 63 | M | ASH | B | A | A0201 | No | □ | □ | ■ | □ |  |  |  | Therapy-naive |
| HCC#29 | 55 | M | HBV/HDV | B | B | A0201 | No | ■ | □ | ■ |  |  |  |  | Theraoy naïve (before TACE) and 2 days and 28 days after TACE |
| HCC#30 | 60 | F | HCV | A | A | A0301 | No |  |  |  |  |  | □ |  | Therapy-naive |
| HCC#31 | 71 | M | ASH | C | B | A0301 | No |  |  |  |  |  | □ |  | Therapy-naive |
| HCC#29 | 55 | M | HBV/HDV | B | B | A0201 | No | ■ | □ | ■ |  |  |  |  | Therapy-naive |
| HCC#30 | 60 | F | HCV | A | A | A0301 | No |  |  |  |  |  | □ |  | Therapy-naive |
| HCC#31 | 71 | M | ASH | C | B | A0301 | No |  |  |  |  |  | □ |  | Therapy-naive |
| HCC#32 | 52 | M | Other | A | B | A0201 | No | ■ | □ |  |  |  |  |  | Therapy-naive (before TACE) and 14 days after TACE |
| HCC#33 | 85 | M | NASH | A | A | A0201 | No | ■ | ■ |  |  |  |  |  | Therapy-naive |
| HCC#34 | 67 | M | ASH | B | C | A0201 | No | ■ | □ |  |  |  |  |  | Therapy-naive |
| HCC#35 | 52 | M | HCV | A | B | A0201 | No | □ | □ |  |  |  |  |  | Therapy-naive |
| HCC#36 | 63 | M | ASH | C | C | A0201 | No | □ | □ |  |  |  |  |  | Therapy-naive |
| HCC#37 | 52 | M | HCV | A | A | A0201 | No | ■ | □ |  |  |  |  |  | Therapy-naive |
| HCC#38 | 80 | F | HCV | A | B | A0201 | No | □ | □ |  |  |  |  |  | Therapy-naive |
| HCC#39 | 65 | M | HCV | A | B | A0201 | No | □ | □ |  |  |  |  |  | Therapy-naive |
| HCC#40 | 62 | F | ASH | A | B | A0201 | No | □ |  |  |  |  |  |  | Therapy-naive |
| HCC#41 | 59 | F | ASH | B | A | A0201 and A0301 | No | □ | □ |  |  |  | □ |  | Therapy-naive |

| Patient ID | Age [years] | Sex | Etiology | Child score | BCLC score | HLA- type | Treatment prior inclusion | CD8+ T-cell response | | | | | | | Phenotypic analysis |
| --- | --- | --- | --- | --- | --- | --- | --- | --- | --- | --- | --- | --- | --- | --- | --- |
|  |  |  |  |  |  |  |  | HLA-A2  MAGE-A3_271-279_ | HLA-A2  NY-ESO-1_157-165_ | HLA-A2  CMV-pp65_495-503_ | HLA-A2  EBV BMLF1_280-288_ | HLA-A2 Influenza M1_58-66_ | HLA-A3  MAGE-A1_96-104_ | HLA-A3  Glypican-3_519-528_ |  |
| HCC#42 | 59 | M | HCV | A | C | A2 and A3 pos. | No | □ | □ |  |  |  | □ |  | Therapy-naive |
| HCC#43 | 64 | M | NASH | A | A | A0201 | No | ■ | □ | ■ | ■ |  |  |  | Corresponding antigen expression |
| HCC#44 | 70 | M | HBV | A | A | A0301 | No |  |  |  |  |  | □ | □ | Corresponding antigen expression |
| HCC#45 | 71 | M | ASH | A | A | A0201 | No | □ | □ |  |  |  |  |  | Corresponding antigen expression |
| HCC#46 | 80 | M | ASH | A | B | A0301 | No |  |  |  |  |  | □ |  | Corresponding antigen expression |
| HCC#47 | 61 | M | HCV | A | A | A0301 | No |  |  |  |  |  | ■ |  | Corresponding antigen expression |
| HCC#48 | 79 | M | ASH | A | B | A0301 | surgery |  |  |  |  |  | □ |  | Corresponding antigen expression |
| HCC#49 | 80 | M | HCV | A | no HCC | A0201 | TACE, surgery | □ |  |  |  |  |  |  | Corresponding antigen expression |
| HCC#50 | 75 | M | ASH | A | B | A2 pos. | surgery, surgery | □ |  |  |  |  |  |  | Corresponding antigen expression |
| HCC#51 | 47 | M | Other | B | n.d. | A2 positive | surgery | ■ |  |  |  |  |  |  | Corresponding antigen expression |
| HCC#52 | 65 | M | Other | A | no HCC | A0201 and A0301 | surgery | □ |  |  |  |  | □ |  | Corresponding antigen expression |

| Patient ID | Age [years] | Sex | Etiology | Child score | BCLC score | HLA- type | Treatment prior inclusion | CD8+ T-cell response | | | | | | | Phenotypic analysis |
| --- | --- | --- | --- | --- | --- | --- | --- | --- | --- | --- | --- | --- | --- | --- | --- |
|  |  |  |  |  |  |  |  | HLA-A2  MAGE-A3_271-279_ | HLA-A2  NY-ESO-1_157-165_ | HLA-A2  CMV-pp65_495-503_ | HLA-A2  EBV BMLF1_280-288_ | HLA-A2 Influenza M1_58-66_ | HLA-A3  MAGE-A1_96-104_ | HLA-A3  Glypican-3_519-528_ |  |
| HCC#53 | 73 | M | ASH | A | B | A0201 | surgery | ■ |  |  |  |  |  |  | Corresponding antigen expression |
| HCC#54 | 59 | F | HCV | A | no HCC | A0201 | surgery | □ |  |  |  |  |  |  | Corresponding antigen expression |
| HCC#55 | 55 | M | HCV | A | B | A3 pos. | No/TACE |  |  |  |  |  | □ | □ | Therapy-naive (before TACE) and 50 days after TACE |
| HCC#56 | 62 | M | ASH | A | B | A3 pos. | No/TACE |  |  |  |  |  | □ | □ | Therapy-naive (before TACE) and 5 days and 34 days after TACE |
| HCC#57 | 51 | F | ASH | B | A | A2 pos. | No/TACE | □ | □ |  |  |  |  |  | Therapy-naive (before TACE) and 2 days and 48 days after TACE |
| HCC#58 | 80 | M | NASH | n.d. | n.d. | A2 pos. | No/TACE | □ | □ |  |  |  |  |  | Therapy-naive (before TACE) and 2 days and 59 days after TACE |
